# [1,2-Bis­(diisopropyl­phosphan­yl)ethane-κ^2^ *P*,*P*′]dichloridonickel(II)

**DOI:** 10.1107/S1600536811021209

**Published:** 2011-06-11

**Authors:** Nahury Y. Castellanos-Blanco, Juventino J. García, Marcos Flores-Alamo

**Affiliations:** a Facultad de Química, Universidad Nacional Autónoma de México, México DF, 04510, Mexico

## Abstract

In the crystal structure of title compound, [NiCl_2_(C_14_H_32_P_2_)], the Ni^II^ atom lies on a twofold rotation axis and shows a slightly distorted square-planar coordination geometry, with a dihedral angle of 10.01 (8)° between the *cis*-Cl—Ni—Cl and *cis*-P—Ni—P planes. There is no significant inter­molecular inter­action except very weak C—H⋯Cl inter­actions. The crystal studied was a racemic twin.

## Related literature

For the synthesis, see: Scott *et al.* (1990 ▶). For applications of nickel complexes to catalytic systems, see: Vicic & Jones (1997 ▶); Arévalo & García (2010 ▶). For related structures, see: Cañavera-Buelvas *et al.* (2011 ▶); Angulo *et al.* (2003 ▶); Dahlenburg & Kurth (2001 ▶).
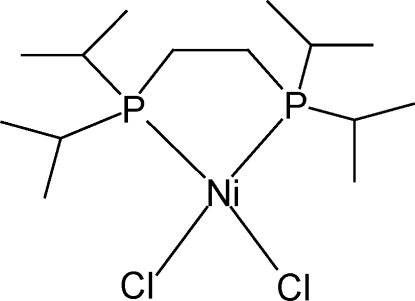


## Experimental

### 

#### Crystal data

[NiCl_2_(C_14_H_32_P_2_)]
*M* *_r_* = 391.95
Tetragonal, 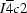

*a* = 14.2402 (2) Å
*c* = 18.4369 (7) Å
*V* = 3738.70 (16) Å^3^
*Z* = 8
Mo *K*α radiation
μ = 1.48 mm^−1^
*T* = 122 K
0.17 × 0.14 × 0.07 mm

#### Data collection

Oxford Xcalibur Atlas Gemini diffractometer
Absorption correction: analytical (*CrysAlis PRO*; Oxford Diffraction, 2010 ▶) *T*_min_ = 0.975, *T*_max_ = 0.989
5823 measured reflections
1850 independent reflections
1547 reflections with *I* > 2σ(*I*)
*R*_int_ = 0.030

#### Refinement

*R*[*F* ^2^ > 2σ(*F* ^2^)] = 0.030
*wR*(*F* ^2^) = 0.070
*S* = 0.97
1850 reflections
92 parameters
H-atom parameters constrained
Δρ_max_ = 0.80 e Å^−3^
Δρ_min_ = −0.23 e Å^−3^
Absolute structure: Flack (1983 ▶), 832 Friedel pairs
Flack parameter: 0.53 (3)

### 

Data collection: *CrysAlis CCD* (Oxford Diffraction, 2009 ▶); cell refinement: *CrysAlis RED* (Oxford Diffraction, 2009 ▶); data reduction: *CrysAlis RED*; program(s) used to solve structure: *SHELXS97* (Sheldrick, 2008 ▶); program(s) used to refine structure: *SHELXL97* (Sheldrick, 2008 ▶); molecular graphics: *ORTEP-3 for Windows* (Farrugia, 1997 ▶); software used to prepare material for publication: *WinGX* (Farrugia, 1999 ▶).

## Supplementary Material

Crystal structure: contains datablock(s) global, I. DOI: 10.1107/S1600536811021209/is2724sup1.cif

Structure factors: contains datablock(s) I. DOI: 10.1107/S1600536811021209/is2724Isup2.hkl

Additional supplementary materials:  crystallographic information; 3D view; checkCIF report

## Figures and Tables

**Table 1:** Selected bond lengths (Å)

Ni1—P1	2.1600 (9)
Ni1—Cl1	2.2150 (8)

**Table 2:** Hydrogen-bond geometry (Å, °)

*D*—H⋯*A*	*D*—H	H⋯*A*	*D*⋯*A*	*D*—H⋯*A*
C3—H3*B*⋯Cl1^i^	0.98	2.94	3.808 (4)	148
C5—H5*A*⋯Cl1^ii^	0.98	2.91	3.777 (4)	148

Symmetry codes: (i) 
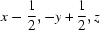
; (ii) 
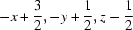
.

**Fig. 1.**
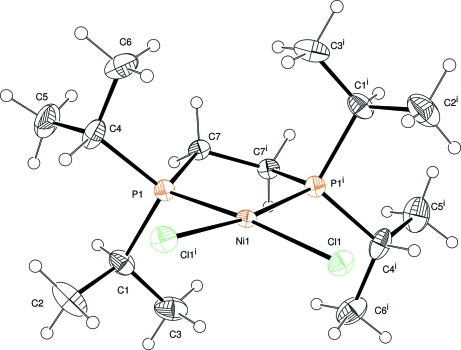
The molecular structure of the title compound. Displacement ellipsoids are drawn at the 50% probability level and H atoms are shown as circles of arbitrary size.

## supplementary crystallographic information

### Comment

The synthesis of the current complex [Ni (dippe)Cl_2_] was documented 21 years ago (Scott *et al.*, 1990) and still the corresponding X-ray structure of this compound has not been reported. This type of nickel complexes are useful starting materials for the preparation of catalysts and catalytic precursors, for a series of active catalyst in a wide variety of model reactions (Vicic & Jones, 1997) and catalytic systems (Arévalo & García, 2010).

In the title complex, [Ni(*dippe*)Cl_2_], the Ni^II^ atom is coordinated by two P atoms of dippe ligand and two chloride anions (Fig. 1) into a slight distorted square-planar coordination geometry with a dihedral angle between the planes defined by the two *cis*-Cl–Ni–Cl and *cis*-P–Ni–P fragments [10.01 (8)°]. Additionally the Ni^II^ atom is situated 0.0837 (1) Å above the Cl1/P1/Cl1/P1 plane. These deviations from planarity, which can be attributed to some steric efect of the dippe ligand, are somewhat shorter than the distortion from ideal square-planar coordination geometry observed on [Ni (dippe)Cl_2_](carbazole)_2_ complex (Cañavera-Buelvas *et al.*, 2011) with the NiCl2/NiP2 dihedral angle of 15.32° and somewhat larger than the distortion from ideal square-planar coordination geometry observed for related [Ni(dcpe)Cl_2_] (Angulo *et al.*, 2003) and [(1*S*,2*S*)-C_5_H_8_{P(C_6_H_11_)_2_}_2_NiCl_2_] (Dahlenburg & Kurth, 2001) complexes, where the NiCl2/NiP2 dihedral angles of 3.96 and 5.37°, respectively.

In the crystal packing, there are two intermolecular contacts of the type hydrogen bond (Table 2) mainly between the carbon donor atom of the dippe to Cl1 chloride atom acceptor of the metallic complex, C5—H5A···Cl1 2.91 Å and C3—H3B···Cl1 2.94 Å.

### Experimental

A concentrated THF solution of the complex [Ni(dippe)Cl_2_], prepared according to the reported procedure (Scott *et al.*, 1990), was stored in a freezer at -30 °C. After a couple of days suitable crystals for X-ray diffraction studies were obtained. NMR (25 °C): ^31^P{^1^H} (CDCl_3_, 121.32 MHz, 25 °C): δ 85.9 (*s*). NMR ^1^H (CDCl_3_,300 MHz, 25 °C): δ 1.30 (m, CH_3_, 24H), 1.6 (m, CH_2_, 4H), 2.48 (m, CH, 4H). Elemental analysis experimental (calculated): C 43.0 (42.9), H 8.24% (8.23%).

### Refinement

H atoms attached to C atoms were placed in geometrically idealized positions, and refined as riding on their parent atoms, with C—H distances fixed to 0.98 (methyl CH_3_), 0.99 (methylene CH_2_) and 1.00 Å (methine CH), and with *U*_iso_(H) = 1.5*U*_eq_(methyl C) or 1.2*U*_eq_(C). The crystal studied was a racemic twin; the minor twin component refined to 47 (3)%.

### Crystal data

**Table:**

[NiCl_2_(C_14_H_32_P_2_)]	*D*_x_ = 1.393 Mg m^−^^3^
*M**_r_* = 391.95	Mo *K*α radiation, λ = 0.71073 Å
Tetragonal, *I*4*c*2	Cell parameters from 3046 reflections
*a* = 14.2402 (2) Å	θ = 3.4–26.0°
*c* = 18.4369 (7) Å	µ = 1.48 mm^−^^1^
*V* = 3738.70 (16) Å^3^	*T* = 122 K
*Z* = 8	Prism, brown
*F*(000) = 1664	0.17 × 0.14 × 0.07 mm

### Data collection

**Table:**

Oxford Xcalibur Atlas Gemini diffractometer	1850 independent reflections
graphite	1547 reflections with *I* > 2σ(*I*)
Detector resolution: 10.4685 pixels mm^-1^	*R*_int_ = 0.030
ω scans	θ_max_ = 26.1°, θ_min_ = 3.4°
Absorption correction: analytical (*CrysAlis PRO*; Oxford Diffraction, 2010)	*h* = −16→17
*T*_min_ = 0.975, *T*_max_ = 0.989	*k* = −17→17
5823 measured reflections	*l* = −22→16

### Refinement

**Table:**

Refinement on *F*^2^	Secondary atom site location: difference Fourier map
Least-squares matrix: full	Hydrogen site location: inferred from neighbouring sites
*R*[*F*^2^ > 2σ(*F*^2^)] = 0.030	H-atom parameters constrained
*wR*(*F*^2^) = 0.070	*w* = 1/[σ^2^(*F*_o_^2^) + (0.0391*P*)^2^] where *P* = (*F*_o_^2^ + 2*F*_c_^2^)/3
*S* = 0.97	(Δ/σ)_max_ = 0.001
1850 reflections	Δρ_max_ = 0.80 e Å^−^^3^
92 parameters	Δρ_min_ = −0.23 e Å^−^^3^
0 restraints	Absolute structure: Flack (1983), 832 Friedel pairs
Primary atom site location: structure-invariant direct methods	Flack parameter: 0.53 (3)

### Special details

**Table:**

Geometry. All s.u.'s (except the s.u. in the dihedral angle between two l.s. planes) are estimated using the full covariance matrix. The cell s.u.'s are taken into account individually in the estimation of s.u.'s in distances, angles and torsion angles; correlations between s.u.'s in cell parameters are only used when they are defined by crystal symmetry. An approximate (isotropic) treatment of cell s.u.'s is used for estimating s.u.'s involving l.s. planes.
Refinement. Refinement of *F*^2^ against ALL reflections. The weighted *R*-factor *wR* and goodness of fit *S* are based on *F*^2^, conventional *R*-factors *R* are based on *F*, with *F* set to zero for negative *F*^2^. The threshold expression of *F*^2^ > 2σ(*F*^2^) is used only for calculating *R*-factors(gt) *etc*. and is not relevant to the choice of reflections for refinement. *R*-factors based on *F*^2^ are statistically about twice as large as those based on *F*, and *R*- factors based on ALL data will be even larger.

### Fractional atomic coordinates and isotropic or equivalent isotropic displacement parameters (Å^2^)

**Table:**

	*x*	*y*	*z*	*U*_iso_*/*U*_eq_	
C1	0.5579 (3)	0.2802 (2)	0.4018 (2)	0.0423 (9)	
H1	0.5088	0.2434	0.3754	0.051*	
C2	0.5856 (3)	0.3616 (3)	0.3527 (3)	0.0708 (15)	
H2A	0.5296	0.3982	0.3403	0.106*	
H2B	0.6144	0.3371	0.3083	0.106*	
H2C	0.6307	0.4019	0.3781	0.106*	
C3	0.5142 (3)	0.3145 (3)	0.4703 (2)	0.0580 (13)	
H3A	0.5614	0.3477	0.4993	0.087*	
H3B	0.49	0.261	0.498	0.087*	
H3C	0.4625	0.3573	0.4588	0.087*	
C4	0.7002 (3)	0.1639 (3)	0.33272 (17)	0.0457 (10)	
H4	0.7238	0.2221	0.3086	0.055*	
C5	0.6214 (3)	0.1240 (3)	0.2847 (2)	0.0676 (12)	
H5A	0.6454	0.1136	0.2355	0.101*	
H5B	0.569	0.1686	0.283	0.101*	
H5C	0.5995	0.0642	0.3049	0.101*	
C6	0.7832 (3)	0.0972 (3)	0.3400 (2)	0.0554 (12)	
H6A	0.7648	0.043	0.3695	0.083*	
H6B	0.8356	0.1299	0.3634	0.083*	
H6C	0.8025	0.0757	0.2918	0.083*	
C7	0.5952 (2)	0.0942 (2)	0.45870 (15)	0.0292 (6)	
H7A	0.5299	0.0924	0.4404	0.035*	
H7B	0.6277	0.0369	0.4416	0.035*	
P1	0.65506 (6)	0.19836 (6)	0.42220 (5)	0.0280 (2)	
Cl1	0.83890 (6)	0.31965 (6)	0.58688 (5)	0.0349 (2)	
Ni1	0.75392 (2)	0.25392 (2)	0.5	0.02082 (13)	

### Atomic displacement parameters (Å^2^)

**Table:**

	*U*^11^	*U*^22^	*U*^33^	*U*^12^	*U*^13^	*U*^23^
C1	0.0335 (19)	0.0391 (19)	0.054 (3)	−0.0022 (16)	−0.0121 (19)	0.0064 (18)
C2	0.053 (3)	0.060 (3)	0.099 (4)	0.003 (2)	−0.009 (3)	0.038 (3)
C3	0.039 (2)	0.050 (2)	0.085 (4)	0.0139 (18)	−0.001 (2)	−0.006 (2)
C4	0.064 (3)	0.046 (2)	0.0270 (18)	−0.019 (2)	0.0057 (17)	−0.0057 (17)
C5	0.090 (4)	0.074 (4)	0.039 (2)	−0.015 (2)	−0.013 (2)	−0.016 (2)
C6	0.067 (3)	0.042 (2)	0.057 (3)	−0.004 (2)	0.022 (2)	−0.013 (2)
C7	0.0251 (18)	0.0270 (19)	0.0355 (16)	−0.0077 (12)	−0.0012 (17)	−0.0046 (17)
P1	0.0269 (4)	0.0268 (4)	0.0303 (5)	−0.0055 (3)	−0.0020 (4)	0.0005 (4)
Cl1	0.0306 (4)	0.0344 (5)	0.0397 (5)	−0.0094 (3)	−0.0042 (4)	−0.0077 (4)
Ni1	0.01688 (15)	0.01688 (15)	0.0287 (3)	−0.00262 (17)	0.00055 (18)	−0.00055 (18)

### Geometric parameters (Å, °)

**Table:**

C1—C3	1.491 (5)	C5—H5A	0.98
C1—C2	1.522 (6)	C5—H5B	0.98
C1—P1	1.847 (3)	C5—H5C	0.98
C1—H1	1.00	C6—H6A	0.98
C2—H2A	0.98	C6—H6B	0.98
C2—H2B	0.98	C6—H6C	0.98
C2—H2C	0.98	C7—C7^i^	1.523 (6)
C3—H3A	0.98	C7—P1	1.838 (3)
C3—H3B	0.98	C7—H7A	0.99
C3—H3C	0.98	C7—H7B	0.99
C4—C6	1.522 (6)	P1—Ni1	2.1600 (9)
C4—C5	1.538 (5)	Cl1—Ni1	2.2150 (8)
C4—P1	1.837 (3)	Ni1—P1^i^	2.1600 (9)
C4—H4	1.00	Ni1—Cl1^i^	2.2150 (8)
			
C3—C1—C2	111.2 (3)	C4—C5—H5C	109.5
C3—C1—P1	110.3 (3)	H5A—C5—H5C	109.5
C2—C1—P1	114.0 (3)	H5B—C5—H5C	109.5
C3—C1—H1	107	C4—C6—H6A	109.5
C2—C1—H1	107	C4—C6—H6B	109.5
P1—C1—H1	107	H6A—C6—H6B	109.5
C1—C2—H2A	109.5	C4—C6—H6C	109.5
C1—C2—H2B	109.5	H6A—C6—H6C	109.5
H2A—C2—H2B	109.5	H6B—C6—H6C	109.5
C1—C2—H2C	109.5	C7^i^—C7—P1	111.27 (13)
H2A—C2—H2C	109.5	C7^i^—C7—H7A	109.4
H2B—C2—H2C	109.5	P1—C7—H7A	109.4
C1—C3—H3A	109.5	C7^i^—C7—H7B	109.4
C1—C3—H3B	109.5	P1—C7—H7B	109.4
H3A—C3—H3B	109.5	H7A—C7—H7B	108
C1—C3—H3C	109.5	C4—P1—C7	105.97 (15)
H3A—C3—H3C	109.5	C4—P1—C1	104.33 (18)
H3B—C3—H3C	109.5	C7—P1—C1	103.68 (17)
C6—C4—C5	112.7 (4)	C4—P1—Ni1	117.79 (14)
C6—C4—P1	111.0 (3)	C7—P1—Ni1	110.76 (10)
C5—C4—P1	111.1 (3)	C1—P1—Ni1	113.10 (12)
C6—C4—H4	107.2	P1—Ni1—P1^i^	87.91 (5)
C5—C4—H4	107.2	P1—Ni1—Cl1	172.36 (3)
P1—C4—H4	107.2	P1^i^—Ni1—Cl1	89.73 (3)
C4—C5—H5A	109.5	P1—Ni1—Cl1^i^	89.73 (3)
C4—C5—H5B	109.5	P1^i^—Ni1—Cl1^i^	172.36 (3)
H5A—C5—H5B	109.5	Cl1—Ni1—Cl1^i^	93.51 (5)
			
C6—C4—P1—C7	−72.2 (3)	C2—C1—P1—C7	−164.7 (3)
C5—C4—P1—C7	54.2 (3)	C3—C1—P1—Ni1	−50.7 (3)
C6—C4—P1—C1	178.8 (3)	C2—C1—P1—Ni1	75.3 (3)
C5—C4—P1—C1	−54.9 (3)	C4—P1—Ni1—P1^i^	−129.91 (14)
C6—C4—P1—Ni1	52.4 (3)	C7—P1—Ni1—P1^i^	−7.74 (12)
C5—C4—P1—Ni1	178.8 (3)	C1—P1—Ni1—P1^i^	108.15 (14)
C7^i^—C7—P1—C4	153.8 (3)	C4—P1—Ni1—Cl1	158.0 (3)
C7^i^—C7—P1—C1	−96.6 (4)	C7—P1—Ni1—Cl1	−79.9 (3)
C7^i^—C7—P1—Ni1	25.0 (4)	C1—P1—Ni1—Cl1	36.0 (3)
C3—C1—P1—C4	−179.9 (3)	C4—P1—Ni1—Cl1^i^	42.82 (14)
C2—C1—P1—C4	−53.9 (4)	C7—P1—Ni1—Cl1^i^	164.99 (13)
C3—C1—P1—C7	69.4 (3)	C1—P1—Ni1—Cl1^i^	−79.12 (14)

Symmetry codes: (i) *y*+1/2, *x*−1/2, −*z*+1.

### Hydrogen-bond geometry (Å, °)

**Table:**

*D*—H···*A*	*D*—H	H···*A*	*D*···*A*	*D*—H···*A*
C3—H3B···Cl1^ii^	0.98	2.94	3.808 (4)	148
C5—H5A···Cl1^iii^	0.98	2.91	3.777 (4)	148

Symmetry codes: (ii) *x*−1/2, −*y*+1/2, *z*; (iii) −*x*+3/2, −*y*+1/2, *z*−1/2.
